# Correlation of lymphocyte-to-monocyte, platelet-to-lymphocyte, and neutrophil-to-lymphocyte ratios with skin-symptom improvement following antimicrobial treatment in palmoplantar pustulosis

**DOI:** 10.1186/s40780-025-00479-6

**Published:** 2025-08-14

**Authors:** Kuninori Iwayama, Takayuki Manabe, Ko-Ichi Ohtaki, Masayuki Chuma, Mari Kishibe, Masaru Honma, Yoshikazu Tasaki

**Affiliations:** 1 Department of Pharmacotherapy & Therapeutics, Faculty of Pharmaceutical Sciences, Hokkaido University of Science, 7-15-4-1 Maeda, Teine, Sapporo, 006-8590 Hokkaido Japan; 2 Department of Hospital Pharmacy & Pharmacology, Asahikawa Medical University, 2-1-1-1 Midorigaokahigashi, Asahikawa, 078-8510 Japan; 3 Department of Nursing, Asahikawa Medical University, 2-1-1-1 Midorigaokahigashi, Asahikawa, 078-8510 Japan; 4 Department of Dermatology, Asahikawa Medical University, 2-1-1-1 Midorigaokahigashi, Asahikawa, 078-8510 Japan; 5 International Exchange Promotion Center, Asahikawa Medical University, 2-1-1-1 Midorigaokahigashi, Asahikawa, 078-8510 Japan

**Keywords:** Palmoplantar pustulosis, Antimicrobial drug treatment, Lymphocyte-to-monocyte ratio, Blood count indicator, Monitoring.

## Abstract

**Background:**

Palmoplantar Pustulosis (PPP) is a chronic inflammatory skin disease that presents as pustules on the palms and soles. There are no oral medications for patients with mild PPP, with apremilast, a new oral PDE4 inhibitor, indicated for moderate to severe cases of the disease. We previously reported that antimicrobial therapy improved PPP symptoms according to dermatological assessment (improvement or not), with a negative correlation between lymphocyte-to-monocyte ratio (LMR) and improvement of symptoms. However, the correlation between PPP area-and-severity index (PPPASI) and LMR could not be evaluated.

**Methods:**

Herein, we used the PPPASI to evaluate the effect of antimicrobial therapy and investigate whether LMR, platelet-to-lymphocyte ratio (PLR), and neutrophil-to-lymphocyte ratio (NLR), which reflect inflammation, could be used as indicators of treatment efficacy. Eleven patients who were administered an antimicrobial agent were enrolled. Treatment efficacy was measured by PPPASI 25 and PPPASI symptom subscales. Treated patients were compared with six control patients who had no change in PPP therapy during the observation period. In addition, the improvement in LMR, PLR, and NLR with antimicrobial treatment was analyzed and correlated with PPPASI.

**Results:**

The antimicrobial drug treatment group was more likely to achieve PPPASI 25 than the control group. PPPASI symptoms subscale for erythema and desquamation were improved compared to those in the control group. There was a correlation between changes in PPPASI correlated with LMR, PLR, and NLR from antimicrobial treatment.

**Conclusions:**

Our results suggest antimicrobial drug treatment as therapy for mild PPP. In addition, LMR, PLR, and NLR are potential biomarkers of antimicrobial drug treatment.

**Trial registration:**

This study is not considered a clinical trial because it is observational.

## Background

Palmoplantar pustulosis (PPP) is a chronic inflammatory skin disease characterized by multiple pustules on the palms and soles, or by pustulotic arthro-osteitis (PAO) pain, in turn having a significant impact on the quality of life of patients [1]. In cases of moderate to severe disease, immunosuppressants and biological agents are used as pharmacological treatment. However, cyclosporin increases the risk of severe infection, renal dysfunction, and hypertension [2–4]. As for biological agents, guselkumab, brodalumab, and risankizumab also cause severe infection. In addition, injectables increase the economic and physical burden on the patient. For mild to moderate PPP, there are few effective systemic drugs; therefore, new drugs are needed [5, 6].

Macrolide and tetracycline antimicrobial agents have anti-inflammatory effects and are used to treat chronic inflammatory respiratory diseases [7–9]. In our previous retrospective study, we found that tetracycline antibiotics improved PPP symptoms and suggested the possibility of lymphocyte-to-monocyte ratio (LMR) as an efficacy biomarker [10]. However, we did not achieve establishment of LMR as an efficacy biomarker because skin-symptoms were roughly evaluated by dermatological assessment as improved or not. In recent years, palmoplantar pustulosis area and severity index (PPPASI) has been used to evaluate the severity of PPP based on erythema, pustules, desquamation, and extent of lesions. However, we could not obtain PPPASI scores because it is not used in daily clinical practice. Therefore, we could not clarify the correlation between PPPASI and LMR.

To clarify the correlation between LMR and severity of PPP symptoms, we performed a prospective study. In addition, we investigated whether PPPASI is correlated with platelet-to-lymphocyte ratio (PLR) or neutrophil-to-lymphocyte ratio (NLR), which are markers of inflammation [11, 12]. These parameters are readily available in clinical practice, cost-effective compared to measurement of inflammatory cytokines, and can be obtained through routine hematological and biochemical examinations. In fact, they have recently been recognized as inflammatory markers in immune-inflammation diseases, including psoriasis and rheumatoid arthritis [13–17]. However, no studies have examined their application in patients with PPP. We hypothesized that the anti-inflammatory effects of antimicrobial drug treatment against PPP were associated with changes in LMR, PLR, and NLR.

In this study, we aimed to assess the efficacy of antimicrobial drug treatment using PPPASI and to investigate whether LMR, PLR, or NLR could be used as efficacy indicators of antimicrobial drug treatment for PPP using PPPASI.

## Methods

### Study design

This was a single center prospective observational study conducted at Asahikawa Medical University Hospital between May 29, 2018, and March 31, 2024. Inclusion criteria encompassed patients starting or being treated with a drug for PPP, age 20 years or older (any gender), and those who could provide written informed consent. Exclusion criteria included patients receiving newly initiated highly effective immunosuppressive agents such as guselkumab or cyclosporine and patients deemed by the physician in charge to be inappropriate for participation in the study.

### Patients and methods

**Fig. 1:**
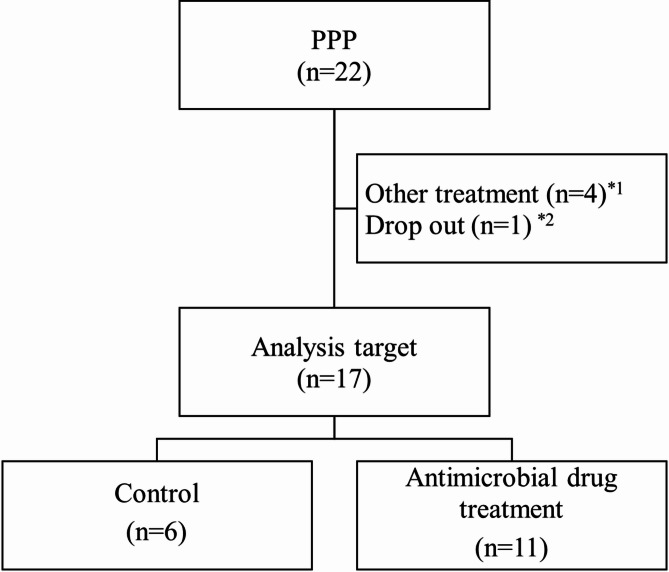
Flow chart

Twenty-two patients gave written consent to participate in the study. Eleven patients were started on antimicrobial therapy with tetracycline or cephem antibiotics as off-label because local topical therapy did not provide sufficient improvement. Six patients continued the same PPP medication during the study period at the Department of Dermatology at Asahikawa Medical University Hospital from June 20, 2018, to May 31, 2022, and were enrolled (Fig. 1). Two of the six patients had been using tetracycline antimicrobials continuously for more than one year before the start of the observation period and maintained the same treatment without any changes throughout the observation period. Therefore, they were not considered new initiators of antimicrobials and were classified as the control group. The observation period was up to 52 weeks from the start date of initiating antimicrobial therapy or follow-up according to our previous report [10]. The end date was defined as the time at which each patient’s blood sample was collected. Blood samples were collected when deemed necessary by the dermatologist. Since our previous paper demonstrated the effectiveness of LMR in treating 10 patients using antimicrobials, this study was conducted with 10 patients in the antimicrobial group. As a result, additional cases were included during the observation period, bringing the overall total to 11.

Informed consent was obtained from 22 patients with PPP. Seventeen patients were included in the analysis set and divided into two groups: control (*n* = 6) and antimicrobial drug treatment (*n* = 11). *1: four patients who had newly initiated treatment with highly effective immunosuppressive agents (such as guselkumab or cyclosporine) were excluded to minimize potential confounding effects of treatment choice on disease severity when comparing outcomes with the antimicrobial treatment group. *2: one patient was excluded due to loss to follow-up after the initial visit.

### Clinical data

Patient characteristics including age, sex, history of cigarette smoking, PAO, focal infection, pharmacotherapy for PPP at the start date, and hematological and biochemical examinations were collected from medical records. White blood cell, platelet (PLT), neutrophil (NEUT), lymphocyte (LYMP), and monocyte (MONO) counts, as well as PLR, NLR, LMR, and C-reactive protein (CRP) levels were collected as hematological and biochemical examination data. PLR was calculated by dividing the PLT count by the LYMP count. NLR was obtained by dividing the NEUT count by the LYMP count. LMR was calculated by dividing the LYMP count by the MONO count. If CRP was < 0.1 mg/dL, the value was treated as 0.1 mg/dL.

#### Efficacy assessment

Efficacy assessment was determined by changes in the PPPASI from the start date using the PPPASI symptom subscale [18, 19]. PPPASI was determined according to the severity and affected area of each symptom on palms and soles. PPPASI symptom subscale only accounted for the severity of each symptom because patients with mild to moderate PPP only develop some of the primary symptoms of erythema, pustules, or desquamation (Table 1). The same dermatologist performed the scoring for one patient. The primary outcomes were the rate of PPPASI 25 and the correlation between PPPASI and LMR as a biomarker. The secondary outcomes were changes in PPPASI subscale scores and correlations between PPPASI and other inflammatory markers, such as NLR and PLR.

**Table 1:** Calculation of palmoplantar pustulosis area and severity index (PPPASI)

Scale	0	1	2	3	4	5	6
Erythema (E)	None	Slight	Moderate	Severe	Very severe		
Pustule (P)	None	Slight	Moderate	Severe	Very severe		
Desquamation (D)	None	Slight	Moderate	Severe	Very severe		
Area affected (%)	0	< 10	10 < 30	30 < 50	50 < 70	70 < 90	90 <

Note: PPPASI = (E + P + D) area affected ×0.2 (right palm) + (E + P + D) area affected ×0.2 (left palm) + (E + P + D) area affected ×0.3 (right sole) + (E + P + D) area affected ×0.3 (left sole), PPPASI ≥ 12 was treated as moderate or high and PPPASI < 12 as mild PPP. Total PPPASI symptom subscale = sum of E, P and D subscale without area affected. PPPASI symptom subscale = one of E, P and D subscale

### Statistical analysis

Continuous parameters including age, clinical laboratory values, PPPASI, and PPPASI symptom subscale are presented as median and min–Max, and qualitative parameters as numbers and percentages. Comparisons of PPPASI and PPPASI symptom subscale for erythema, pustules, and desquamation between the start and end dates were analyzed using the paired *t*-test or Wilcoxon signed-rank test after normality distribution and equal variances were checked in efficacy assessment. Comparisons of PPP improvement between antimicrobial drug treatment and control groups were determined using Fisher’s extract test, unpaired *t*-test, and Mann–Whitney U test. Statistical analyses were performed using GraphPad Prism ver. 10.0.4 (GraphPad Software, San Diego, CA, USA). To assess the relationship between the PPPASI and clinical laboratory data after treatment, the interlock coefficient (*λ*) was calculated using the change in PPPASI and clinical laboratory data from the start date [20]. In all analyses, differences were considered statistically significant at *P* < 0.05.

### Ethical approval

The study protocol and informed consent forms were in accordance with the Declaration of Helsinki and its amendments and the ethical guidelines for medical and health research involving human subjects. Written informed consent was obtained from all participants before commencement of the study. Ethical approval was obtained from the Asahikawa Medical University Research Ethics Committee (approval no. 18006).

## Results

### Baseline characteristics of patients with PPP

Patients in the antimicrobial drug treatment group were younger than those in the control group (*P* = 0.02). In addition, the antimicrobial drug group had an earlier age of disease onset (*P* = 0.047). There was no difference in history of smoking, which is an exacerbating factor of PPP, between the control and the antimicrobial drug treatment groups. Other patient characteristics and severity of PPP, PPPASI, and symptom subscale did not differ between the two groups (Table 2). Eight patients received tetracycline (500 or 1000 mg/day), one received minocycline (100 mg), one received doxycycline (100 mg), and one received cefcapene peroxyl hydrochloride (300 mg) as part of antimicrobial drug treatment. The median of administration term was 176 days [min–Max: 45–357].

**Table 2:** Study population characteristics at initiation of antimicrobial drug treatment

	Control (*n* = 6)	Antimicrobial drug treatment (*n* = 11)	*P* value
**Age (years)**,** median [min- Max]**	**62 [45–74]**	**52 [33–64]**	**0.02** ^**a)**^
Duration of disease (years), median [min- Max]	7.6 [0.2–28.5]	4.5 [0–34.3]	0.92^a)^
**Age at onset (years)**,** median [min- Max]**	**52 [42–60]**	**39 [23–56]**	**0.047** ^**a)**^
Female, n (%)	6 (100%)	9 (81.8%)	0.51
Smoking, n (%)	5 (83.3%)	7 (63.6%)	0.60
PPPASI, median [min- Max]	1.8 [0–13.2]	4.3 [0.6–15.6]	0.19 ^b)^
Total PPPASI symptom subscale, median [min- Max]	3 [0–7]	6 [2–7]	0.10 ^a)^
PAO, n (%)	4 (66.7%)	7 (63.6%)	1.00
Focal infection, n (%)	2 (33.3%)	5 (45.5%)	1.00
Concomitant drug, n (%)			
Topical steroid	2 (33.3%)	7 (63.6%)	0.33
Topical vitamin D_3_	3 (50.0%)	3 (27.3%)	0.60
Biotin	2 (33.3%)	7 (63.6%)	0.33
NSAIDs	1 (16.7%)	4 (36.4%)	0.60
Immunosuppressants	0	1 (9.1%)	1.00
Biological drug	1 (16.7%)	0	0.35
Clinical laboratory data, median [min – Max]			
WBC (/µL)	6600 [5810–7980]	5760 [4220–9190]	0.88^b)^
PLT (×10^3^/µL)	233 [163–331]	280 [190–360]	0.48 ^a)^
NEUT (/µL)	4085 [3200–5090]	3500 [2440–6360]	1.00 ^a)^
LYMP (/µL)	2045 [1540–2440]	1480 [1210–3960]	0.07 ^b)^
MONO (/µL)	280 [270–370]	340 [240–680]	0.27 ^b)^
PLR	113 [70–215]	162 [87–245]	0.19 ^a)^
NLR	2.08 [1.37–2.83]	1.97 [0.96–3.65]	0.40 ^a)^
LMR	6.65 [4.16–9.04]	4.35 [2.95–8.43]	0.07 ^a)^
CRP (mg/dL)	0.10 [0.10–0.41]	0.10 [0.10–0.31]	0.95 ^b)^

Note: Quantitative variables are presented as medians (min–Max), and qualitative variables as counts (percentages). Drug treatment is expressed as use at start date, including prescriptions from other hospitals. (a) unpaired *t*-test and (b) Mann–Whitney U test

*Abbreviations*: PPPASI, PPP area and severity index; PAO, pustulotic arthroosteitis; VAS, visual analog scale; WBC, white blood cells; PLT, platelets; NEUT, neutrophils; LYMP, lymphocytes; MONO, monocytes; PLR, platelet-to-lymphocyte ratio; NLR, neutrophil-to-lymphocyte ratio; LMR, lymphocyte-to-monocyte ratio; CRP, C-reactive protein

### Antimicrobial drug treatment decreases erythema and desquamation severity

**Fig. 2:**
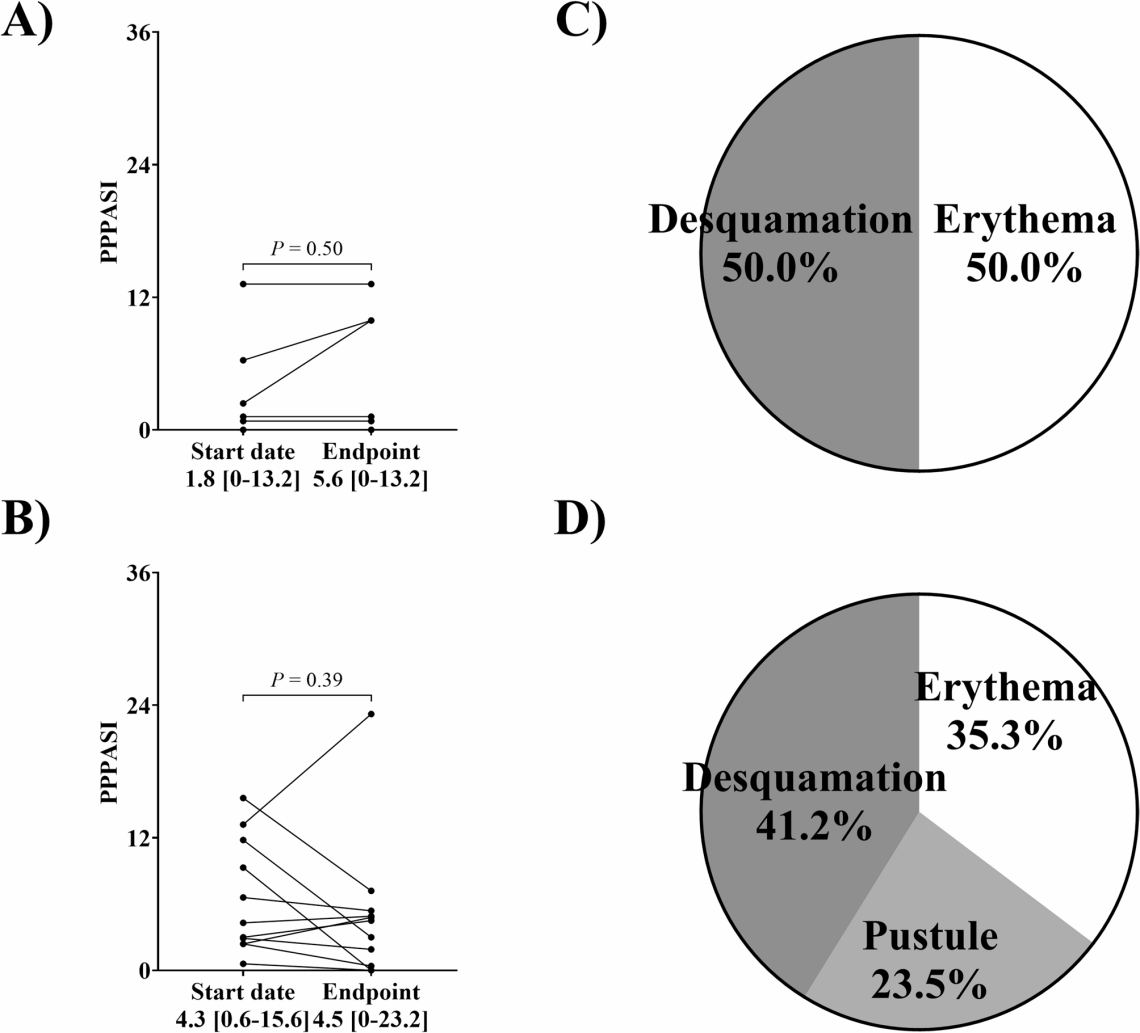
Change in PPPASI of the most severe skin lesion from the start date. The change in PPPASI in (**A**) the control group (*n* = 6) and (**B**) the antimicrobial treatment group (*n* = 11). PPPASI is calculated as the sum of the severity of erythema, pustules, and desquamation, with a correction for the area and site involved (palm or sole). The most severe skin lesion (**C**) in the control group (*n* = 6) and (**D**) antimicrobial drug treatment group (*n* = 11). Patients had multiple disease severity lesions. Statistical analyses were performed using a Wilcoxon signed-rank test for (**A**) or a paired t-test for (**B**) between start and end dates. Differences were considered statistically significant at *P* < 0.05

The efficacy of antimicrobial agents against PPP was evaluated using PPPASI 25, 50, 75, and 100. PPPASI 25 was defined as a greater than 25% improvement in PPPASI from start date to end date. There was no observation of PPPASI improvement in the control group. Contrarily, antimicrobial drug treatment achieved PPPASI 25, PPPASI 50, PPPASI 75, and PPPASI 100 in 54.5%, 45.5%, 36.4%, and 18.2% of patients, respectively (Table 3). There was a significant difference between control and antimicrobial drug treatment in PPPASI 25 (*P* = 0.04). However, there was no significant difference between the start date and the end date in the change in PPPASI in both groups (Fig. 2A and B). Next, we focused our evaluation on the most severely affected area, as there are different types of patients with symptomatic PPP. In the control group, the severity of the disease was in the erythema and desquamation (Fig. 2C). In the antimicrobial drug treatment group, it was located in the desquamation (Fig. 2D). Therefore, we evaluated the groups according to each symptom subscale and total symptoms.

**Table 3:** Achievement of PPP improvement rate at end date

	Control (*n* = 6)	Antimicrobial drug treatment (*n* = 11)	*P* value
**PPPASI 25**,** n (%)**	**0**	**6 (54.5%)**	**0.04**
PPPASI 50, n (%)	0	5 (45.5%)	0.10
PPPASI 75, n (%)	0	4 (36.4%)	0.24
PPPASI 100, n (%)	0	2 (18.2%)	0.51

Note: Qualitative variables as counts (percentages)

*Abbreviations*: PPPASI, PPP area and severity index. PPPASI 25, PPPASI ≧ 25% improvement at end date. PPPASI 50, PPPASI ≧ 50% improvement at end date. PPPASI 75, ≧75% improvement at end date. PPPASI 100, 100% improvement in PPPASI at end date

**Fig. 3:**
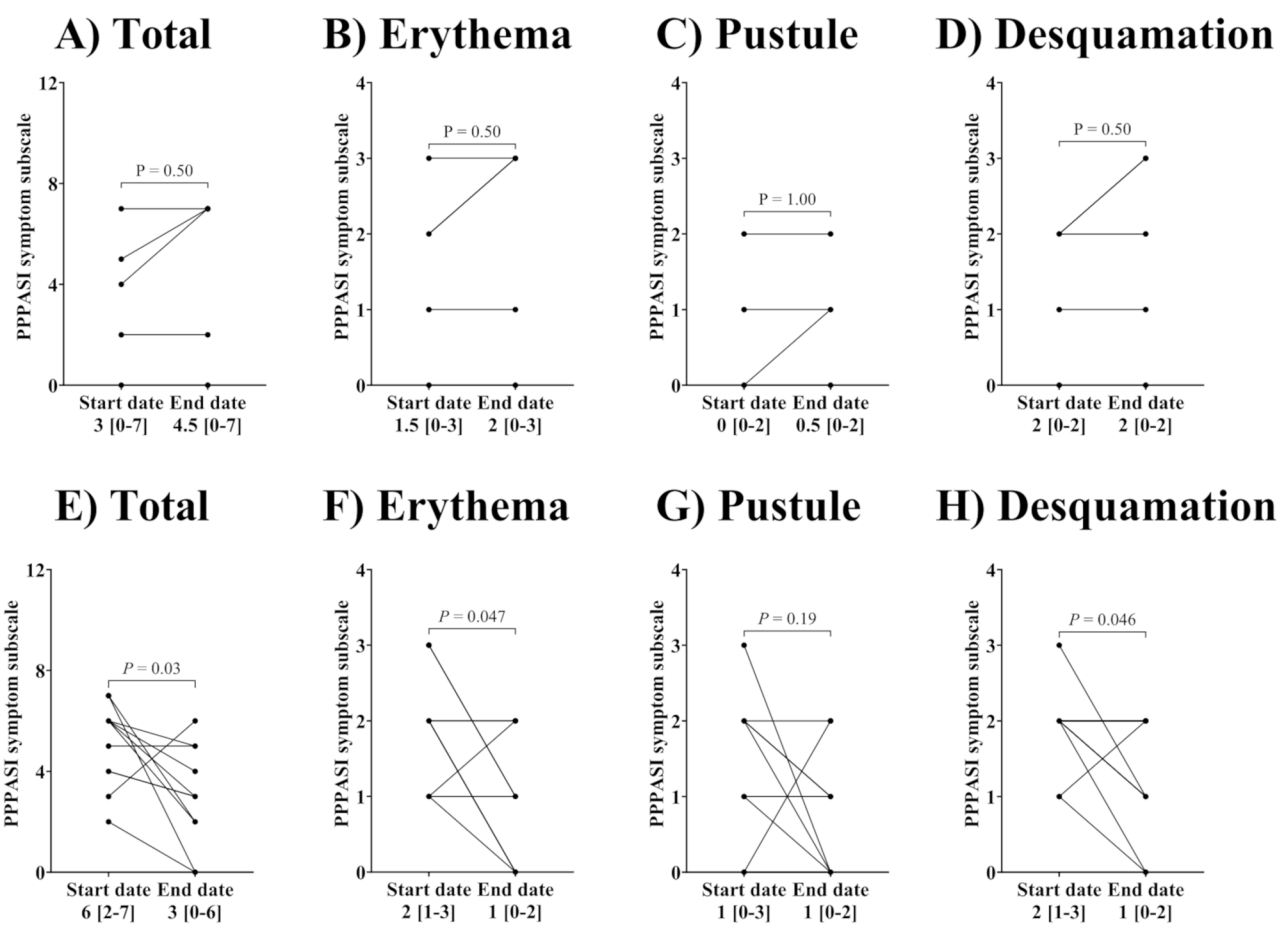
Change in the PPPASI subscale for erythema, pustules, and desquamation after antimicrobial drug treatment. Control group showing (**A**) total PPPASI symptom subscale, (**B**) erythema, (**C**) pustules, and (**D**) desquamation (*n* = 6). Effects of antimicrobial drug treatment in (**E**) total PPPASI symptom subscale, (**F**) erythema, (**G**) pustules, and (**H**) desquamation (*n* = 11). Total PPPASI symptom subscale was calculated as the sum of erythema, pustules, and desquamation severity. The PPPASI subscales were categorized as follows: 0; none, 1; slight, 2; moderate, 3; severe, 4; very severe. Data are expressed as median [min – Max]. Statistical analyses were performed using a Wilcoxon signed-rank test for **A**)–**D**) and **F**) or a paired t-test for **E**), **G**)–**H**) between start and end dates. Differences were considered statistically significant at *P* < 0.05

**Fig. 4:**
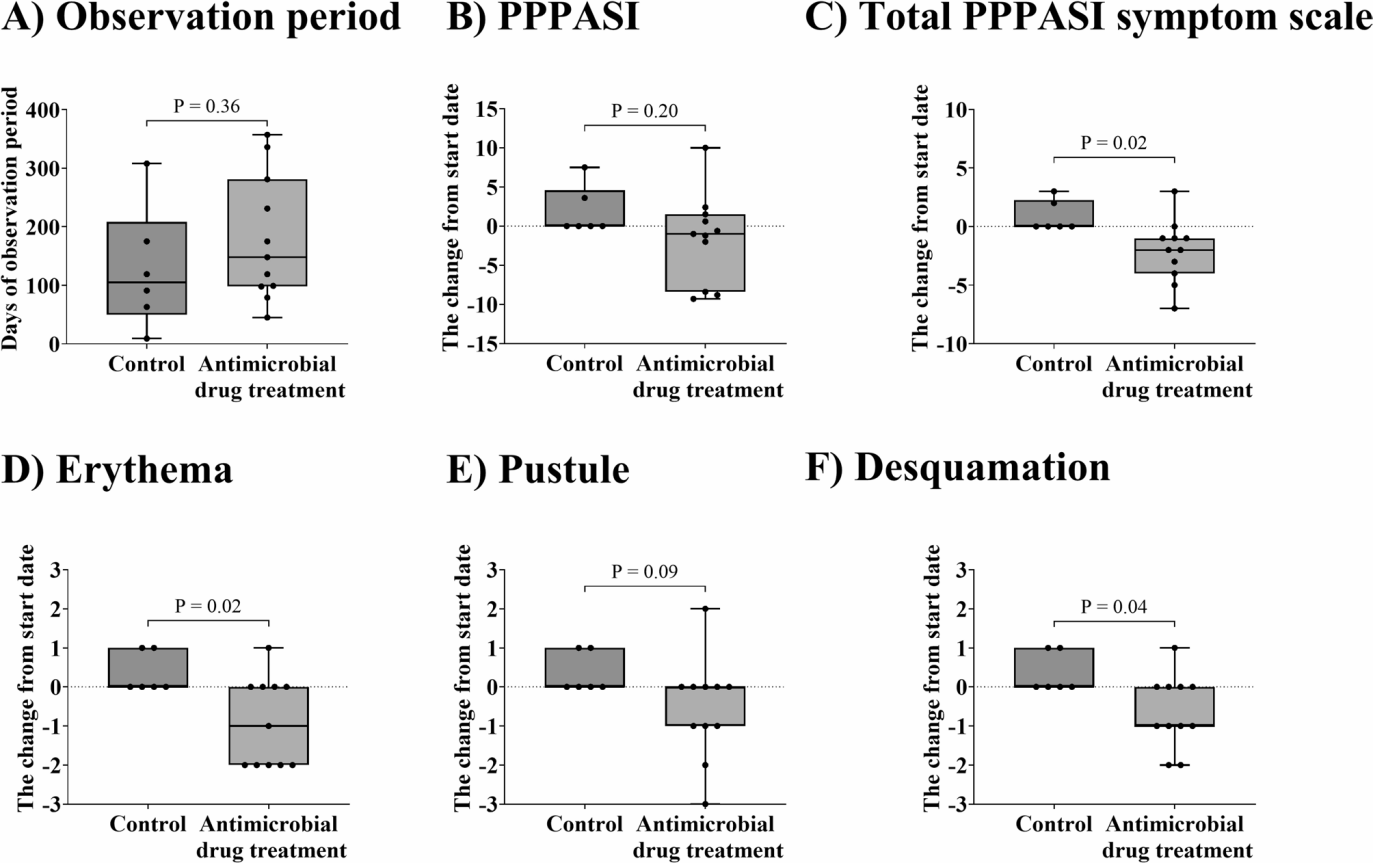
Comparison of change in PPPASI, PPPASI subscale of erythema, pustules, and desquamation between control and antimicrobial drug treatment groups. Comparison of change in (**A**) observation period, (**B**) PPPASI, (**C**) total PPPASI symptom scale, (**D**) erythema, (**E**) pustules, and (**F**) desquamation from start date between the control and antimicrobial drug treatment groups. Total PPPASI symptom subscale values were calculated as the sum of erythema, pustules, and desquamation severity. The PPPASI subscale values were categorized as follows: 0; none, 1; slight, 2; moderate, 3; severe, 4; very severe. Data are expressed as median [min – Max]. Statistical analyses were performed using a paired t-test for **A**)–**C**) or a Wilcoxon signed-rank test for **D**)–**F**) between the control and antimicrobial drug treatment groups. Differences were considered statistically significant at *P* < 0.05

There was no difference in the total PPPASI symptom subscale, the sum of erythema, pustules, and desquamation, and each symptom scale in the control group (Fig. 3A–D). In contrast, antimicrobial drug treatment decreased total symptoms significantly from 6 [2–7] to 3 [0–6] (Fig. 3E, *P* = 0.03). For each symptom, the PPPASI subscale for erythema and desquamation decreased from the start date after antimicrobial drug treatment (Fig. 3F and H, *P* = 0.047 and *P* = 0.046, respectively). However, no difference was observed for pustules (Fig. 3G). In addition, the change in each parameter from the start date was compared between the two groups (Fig. 4). There were differences between the control and antimicrobial drug treatment groups in the total PPP symptom scale (*P* = 0.02, Fig. 4C), erythema (*P* = 0.02, Fig. 4D), and desquamation (*P* = 0.04, Fig. 4F).

### Changes in LYMP levels or PLR, NLR, and LMR are associated with PPPASI in antimicrobial drug treatment

To assess whether the efficacy of antimicrobial treatment against PPP can be monitored using clinical laboratory values, we calculated the interlocking coefficient between the change in PPPASI and each clinical laboratory value. Positive correlation was observed for ΔLYMP (λ = 0.79, *P* < 0.01) and ΔLMR (λ = 0.86, *P* < 0.01), whereas ΔPLR (λ= -0.60, *P* = 0.04) and ΔNLR (λ= -0.63, *P* = 0.03) showed negative correlation (Table 4). Contrarily, no correlation was observed in the control group.

**Table 4:** Interlock coefficients between the change in PPPASI and clinical laboratory data

ΔPPPASI	Control (*n* = 6)		Antimicrobial drug treatment (*n* = 11)	
	λ	*P* value	λ	*P* value
ΔWBC (/µL)	-0.39	0.39	0.01	0.98
ΔPLT (×10^3^/µL)	0.59	0.17	-0.04	0.89
ΔNEUT (/µL)	-0.30	0.52	-0.33	0.29
ΔLYMP (/µL)	-0.60	0.15	**0.79**	**< 0.01**
ΔMONO (/µL)	-0.75	0.050	-0.44	0.15
ΔPLR	0.57	0.18	**-0.60**	**0.04**
ΔNLR	-0.01	0.98	**-0.63**	**0.03**
ΔLMR	0.59	0.17	**0.86**	**< 0.01**
ΔCRP (mg/dL)	0	1.00	-0.13	0.68

Note: Bold values indicate statistically significant findings (*P* < 0.05)

*Abbreviations*: Δ, change in each parameter from the start date to end date; λ, interlock coefficient; WBC, white blood cell; PLT, platelet; NEUT, neutrophil; LYMP, lymphocyte; MONO, monocyte; PLR, platelet-to-lymphocyte ratio; NLR, neutrophil-to-lymphocyte ratio; LMR, lymphocyte-to-monocyte ratio; CRP, C-reactive protein

## Discussion

We present two new findings from this prospective study. First, antimicrobial treatment achieved PPPASI 25 and improved total and two PPPASI symptom subscales compared with the control group. Second, PLR, NLR, and LMR were useful indicators of PPPASI following antimicrobial drug treatment. Based on these findings, we were able to pursue our previously reported retrospective hypothesis.

Antimicrobial drug treatment achieved PPPASI 25 and improved total PPPASI symptom subscale, suggesting usefulness in PPP treatment. Due to the limited number of PPP cases, the power calculation was based on the PPPASI 25 achievement obtained in this study; six control cases showed no improvement, and 11 antimicrobial-treated cases showed 54.5% improvement, resulting in a power of 0.679. Because this study had a limited number of cases, we believe that further clinical studies are needed to demonstrate the efficacy of antimicrobial agents. The PPPASI was not improved in antimicrobial treatment group (Fig. 2B). This could be attributed to the following: in the case of mild PPP, the PPPASI is small, making it difficult to differentiate between treatment effects; the PPPASI is calculated from the severity of three symptoms (desquamation, erythema, pustules) and the area of the lesion, but different patients have different areas of severity, causing variation in the evaluation (Fig. 2C and D). When we focused on the most severely affected site in each patient and evaluated the changes before and after antimicrobial administration, we observed that antimicrobial treatment reduced erythema and desquamation severity scores by the end date compared to the start date (Fig. 3F and H). In addition, these symptoms also improved on the PPP symptom scale with antimicrobial administration compared to the control group (Fig. 4C, D, and F). We believe that the lack of improvement in the pustules was because more than half of the cases were mildly symptomatic. We hypothesize that inflammatory cytokines are involved in all symptoms, including pustules, erythema, and desquamation, and that antimicrobial agents suppress these cytokines and are also involved in lymphocyte regulation [21–23]. Unlike macrolides and tetracyclines, cephem antibiotics are generally not considered to have anti-inflammatory properties. One patient treated with cefcapene peroxyl hydrochloride (CFPN-PI), a cephem antibacterial agent, was included, and in a previous report, CFPN-PI improved PPP in some cases. Since the main objective of this study was to evaluate the efficacy of antimicrobial agents, these agents were included in the analysis regardless of their anti-inflammatory effects. Therefore, antimicrobial therapy may be a treatment option for mild PPP. Therefore, antimicrobial therapy may be a treatment option for mild PPP. Although PPP is associated with focal infections, we do not believe the improvement in erythema and desquamation observed in this study was a consequence of antimicrobial treatment. There was no improvement in PPPASI in patients with focal infections who received antimicrobial therapy (data not shown). In this prospective study, there was a difference in the age of patients with PPP between the control and antimicrobial drug treatment groups (Table 2). However, there was no difference in the proportion of elderly patients (over 65 years old) between the two groups (data not shown). Patients in this study were more likely to be women and smokers, consistent with the ratio in previous reports, respectively [24, 25].

Among the biomarkers, LYMP levels, PLR, NLR, and LMR reflected changes following antimicrobial therapy (Table 4). In particular, LMR was consistent with the results of a previous retrospective study, suggesting its utility as a biomarker [10]. Therefore, the correlation between the LMR and PPPASI suggests that LMR can be used to evaluate the efficacy for PPP. The use of this method would reduce the workload of dermatologists during PPP consultations. LYMPs are also associated with PPPASI in antimicrobial therapy; they are an important component in the development and exacerbation of autoimmune disease and the production of inflammatory cytokines [26]; therefore, herein, it is possible that the improvement in inflammation by antimicrobial drug treatment may have reduced LYMPs. In addition, PLR and NLR, which reflect local inflammation, may also be useful indicators of antimicrobial treatment [11, 27]. As these indicators were not observed in our retrospective study, further investigation is required [10]. We believe that no significant difference in PPPASI change was observed because most of the subjects in this study were mild cases and some of them had exacerbations after antimicrobial administration. However, we suppose that clinical changes were observed because improvement was observed when focusing on the severity of each symptom. In biomarker analysis, the interpretation of correlations was considered appropriate because the results of exacerbations and improvements were consistent. This study has several limitations. First, the sample size was small. Consequently, the control group included cases in which antimicrobial treatment had been initiated more than one year prior to the start of the observation period, with the same dosage maintained throughout the observation. In the future, it may be necessary to increase the number of control cases without antimicrobial treatment for more robust analysis. Nonetheless, despite the limited number of cases, we were able to establish evaluation criteria based on our previous study. Second, due to the observational nature of the study, the blood collection points were inconsistent. We believe that the limitations of this study can be addressed by conducting future interventional studies.

## Conclusions

In conclusion, antimicrobial treatment can be an effective pharmacotherapy for mild PPP. In addition, LMR can be used as an indicator of antimicrobial efficacy in the treatment of PPP, in turn reducing the burden of evaluation of PPP by dermatologists. In addition, PLR and NLR are potential biomarkers for antimicrobial therapy. Overall, we believe that antimicrobial therapy may be a potential strategy for managing PPP.

## Data Availability

The datasets generated and analyzed during the current study are not publicly available due patient privacy but are available from the corresponding author on reasonable request.

## Abbreviations

CRP: C-reactive protein
LYMP: Lymphocyte
LMR: Lymphocyte-to-monocyte ratio
MONO: Monocyte
NEUT: Neutrophil
NLR: Neutrophil-to-lymphocyte ratio
PPP: Palmoplantar Pustulosis
PPPASI: Palmoplantar pustulosis area and severity index
PLT: Platelet
PLR: Platelet-to-lymphocyte ratio
PAO: Pustulotic arthro-osteitis
